# Recent genetic findings in schizophrenia and their therapeutic relevance

**DOI:** 10.1177/0269881114553647

**Published:** 2015-02

**Authors:** Paul J Harrison

**Affiliations:** University Department of Psychiatry, Warneford Hospital, Oxford, UK

**Keywords:** Genomics, GWAS, psychosis, target identification, target validation, *ZNF804A*

## Abstract

Over 100 loci are now associated with schizophrenia risk as identified by single nucleotide polymorphisms (SNPs) in genome-wide association studies. These findings mean that ‘genes for schizophrenia’ have unquestionably been found. However, many questions remain unanswered, including several which affect their therapeutic significance. The SNPs individually have minor effects, and even cumulatively explain only a modest fraction of the genetic predisposition. The remainder likely results from many more loci, from rare variants, and from gene–gene and gene–environment interactions. The risk SNPs are almost all non-coding, meaning that their biological significance is unclear; probably their effects are mediated via an influence on gene regulation, and emerging evidence suggests that some key molecular events occur during early brain development. The loci include novel genes of unknown function as well as genes and pathways previously implicated in the pathophysiology of schizophrenia, e.g. NMDA receptor signalling. Genes in the latter category have the clearer therapeutic potential, although even this will be a challenging process because of the many complexities concerning the genetic architecture and mediating mechanisms. This review summarises recent schizophrenia genetic findings and some key issues they raise, particularly with regard to their implications for identifying and validating novel drug targets.

## Introduction

It has long been known that schizophrenia has a substantial genetic component, with a complex, non-Mendelian inheritance. Estimates of heritability range from ~65–80% (Lichtenstein et al., 2009; Sullivan et al., 2003). Recent research has considerably advanced our understanding in terms of identifying risk loci, the nature of the genetic architecture, and the mechanisms by which genetic risk is conferred (Giusti-Rodriguez and Sullivan, 2013; Gratten et al., 2014; Mowry and Gratten, 2013). Equally, the data emphasise just how complicated is the picture, how little of it has yet been revealed, and the challenges that remain in translating the information into clinically or therapeutically relevant advances (McCarroll and Hyman, 2013; Muglia, 2012; O’Connell et al, 2011). Here, the main findings and themes to emerge from the recent genomic studies of schizophrenia are briefly summarised, before considering their therapeutic implications in terms of target discovery and validation.

## The genetic architecture of schizophrenia

Genetic risk for schizophrenia arises from different forms of DNA sequence variation: the best established are those due to single nucleotide polymorphisms (SNPs) and copy number variants (CNVs). Both act as risk factors; there are no confirmed causal mutations, nor families in which schizophrenia segregates in a Mendelian fashion.

### SNPs

Genome-wide association studies (GWAS) have yielded, as their sample size has grown, increasing and now unequivocal evidence for common SNPs contributing to schizophrenia risk. In a study and meta-analysis involving about 21,000 cases and 38,000 controls by the Psychiatric Genetics Consortium (PGC) (Ripke et al., 2013), 22 loci were identified which contain SNP(s) genome-wide significant for association to schizophrenia. These can be considered statistically robust and indicate that one or more genes at the locus, and one or more variant within the gene(s), contribute to schizophrenia risk (Table 1). These SNPs are but the tip of the iceberg, with estimates that over 8000 SNPs independently contribute to schizophrenia, and which together will explain over 50% of the genetic predisposition (Ripke et al., 2013). The findings confirm that schizophrenia is a highly polygenic disorder (Lee et al., 2012; Purcell et al., 2009). A new analysis from the PGC on a considerably enlarged sample (‘PGC2’, in total, almost 37,000 cases and 113,000 controls) has begun to reveal these additional genes, identifying over 100 loci (implicating about 600 genes) that are now genome-wide significant for schizophrenia (Schizophrenia Working Group of the Psychiatric Genomics Consortium, 2014).

**Table 1.** Selected loci and genes showing association to schizophrenia: nomenclature and notes.

Locus	Implicated gene	Name of gene/product	Notes	Reference to gene biology
12p13.33	*CACNA1C*	L-type calcium channel α subunit, type 1c (Ca_v_1.2)	Important in neuronal function. Mutations cause Timothy syndrome and Brugada syndrome.	Bhat et al., 2012
12q24.11	*DAO*	D-amino acid oxidase	Enzyme which degrades the NMDA receptor co-agonist D-serine. Expression and activity increased in schizophrenia. Not GWAS significant.	Verrall et al., 2010
1q42.2	*DISC1*	Disrupted in schizophrenia-1	Identified in a large Scottish pedigree with a chromosome 1:11 translocation. A multifunctional scaffolding protein. Not GWAS significant.	Brandon and Sawa, 2011
11q23.2	*DRD2*	Dopamine D2 receptor	Long known to be the key target of antipsychotic drugs, GWAS data now indicate that the *DRD2* gene may play a role in schizophrenia.	Beaulieu and Gainetdinov, 2011
2q33-34	*ERBB4*	Receptor tyrosine kinase erbB4	Receptor for neuregulin 1 and some other ligands. Mutations can cause cancers. Not GWAS significant.	Mei and Xiong, 2008
5q33.2	*GRIA1*	AMPA receptor subunit 1 (GluA1; GluR1)	The subunit influences properties of the AMPA receptor, and affects synaptic plasticity and behaviour.	Barkus et al., 2014
16p13.2	*GRIN2A*	NMDA receptor subunit 2A (GluN2A; NR2A)	The subunit influences properties of the NMDAR, including synaptic localisation and channel conductance.	Paoletti et al., 2013
7q21.11-12	*GRM3*	Metabotropic glutamate receptor 3 (mGlu3)	Group II metabotropic glutamate receptor (along with mGlu2), acting primarily as inhibitory autoreceptors.	Harrison et al., 2008
1p21.3	*MIR137*	MicroRNA 137	Non-protein-coding gene. A microRNA, which regulates other genes by binding to the 3’ untranslated region of their transcripts.	Pasquinelli, 2012
8p12	*NRG1*	Neuregulin 1	Growth factor, involved in many aspects of nervous system development and plasticity. Not GWAS significant.	Mei and Nave, 2014
17p13.3	*SRR*	Serine racemase	Enzyme which synthesises D-serine from L-serine.	Balu et al., 2013
18q21.2	*TCF4*	Transcription factor 4	Basic helix-loop-helix transcription factor. Haploinsufficiency causes Pitt–Hopkins syndrome.	Forrest et al., 2014
2q32.1	*ZNF804A*	Zinc finger protein 804A	Putative transcription factor. See Box.	Hess and Glatt, 2013

The table includes the genes mentioned in this article. It is not an exhaustive list, and CNVs are not included. One or more SNPs at each locus are GWAS significant for schizophrenia unless stated.

Several factors are important to note about the GWAS findings:

1. The associations are to genomic regions (‘loci’), not to genes. For most if not all the loci, it is not certain which gene is affected, since the loci often encompass more than one gene; a SNP can regulate expression of genes either upstream or downstream within – or outside – the locus; and SNPs can impact on non-protein-coding genes (or unknown genes) as well as annotated protein-coding genes (Maurano et al., 2012). It is a particular problem at the major histocompatibility complex (MHC) locus (see below). Thus, the genes listed in Table 1 – and the estimate of 600 genes implicated within the 108 loci itself – should be viewed with caution.
2. Virtually all the schizophrenia-associated SNPs are in non-coding regions of DNA (e.g. intergenic, or intronic) or are synonymous exonic polymorphisms. Moreover, there is usually no prior evidence for functional differences between the risk and non-risk alleles. This makes identifying the biological basis for – and the therapeutic potential of – the genetic association extremely challenging. This difficulty is exacerbated by the fact that the measured SNP is unlikely to be the causative SNP at the locus, but is simply tagging a length of DNA within which resides the causal variant or variants (Chakravarti et al., 2013; Edwards et al., 2013; Kircher et al., 2014; Schaub et al., 2012). The scale of the problem is highlighted by Need and Goldstein (2014), who note that only about 20 out of 7300 GWAS associations with common diseases and traits have been clearly tracked to a causal variant. One possibility is that the ‘true’ variant *is* coding (i.e. does change the amino acid sequence of the encoded protein); however, this is not the case for the schizophrenia genes investigated so far. Instead, the most likely explanation is that the risk SNP alters gene regulation, affecting transcription or other events which together determine the abundance, timing, or location, of expression of the gene. There is emerging evidence to support this assumption for at least some schizophrenia genes (Kleinman et al., 2011); in several instances the SNP effect is quite specific, modulating expression of a specific isoform by biasing alternative promoter usage or splicing of the gene (e.g. Law et al., 2006, 2007; Sartorius et al., 2008; Tao et al., 2014); albeit such studies cannot prove that this is the mechanism of disease risk (nor that the investigated SNP is the causal one).
3. The odds ratios associated with each schizophrenia risk SNP are typically around 1.10 and rarely exceed 1.20. Hence each one has a very small effect on disease risk. This finding has important interpretational and investigational implications (see below).
4. Some of the SNPs also show association to broader phenotypes including bipolar disorder and, to a lesser extent, major depression, attention-deficit hyperactivity disorder, and autism (Cross Disorder Group of the Psychiatric Genomics Consortium, 2013a). These findings, an illustration of genetic pleiotropy (Solovieff et al., 2013), suggest that the clinical overlap between these disorders arises at least partly from a shared genetic predisposition (Cross Disorder Group of the Psychiatric Genomics Consortium, 2013b).
5. It is not known whether different schizophrenia risk genes are associated with different subtypes or clinical features of the disorder, in part because of the limited phenotyping of the subjects included in GWAS studies (Bergen et al., 2014). However, initial data suggest that some genes may be associated with cognition in patients (Walters et al., 2010), or with a younger age of onset (Lett et al., 2013; but see Wang et al., 2011).
6. The initial GWAS studies, including Ripke et al. (2013), failed to replicate convincingly any of the genes which had been identified from the pre-GWAS era (i.e. from ‘candidate gene’ approaches), even the seemingly robust findings (Allen et al., 2008; Sullivan et al., 2012). This contributed to controversy about the extent to which genome-wide statistical significance is a *sine qua non* when deciding if a gene is or is not a risk gene, or whether other kinds of evidence (notably biological findings) should also be taken into account (see Abbott, 2008); this issue is best exemplified by debate about *DISC1* (Porteous et al., 2014; Sullivan, 2013). In any event, the PGC2 analysis (Schizophrenia Working Group of the Psychiatric Genomics Consortium, 2014) does implicate several candidate genes, with genome-wide significant findings for loci containing the dopamine D2 receptor (*DRD2* – the ultimate schizophrenia candidate gene) and several glutamate receptors (*GRIN2A, GRIA1, GRM3*). In the case of *GRIN2A* and *GRM3*, the GWAS locus is intragenic, increasing the likelihood that it is the gene itself which underlies the association (see point 1 above). The GWAS support for some candidate genes is therapeutically relevant since the latter were often chosen for study because of their known or hypothesised role as drug targets.

Some of these genetic considerations are discussed further below, in the context of their psychopharmacological implications. The Box illustrates in more detail some of the key aspects about SNP associations to schizophrenia, taking the example of *ZNF804A*, arguably the first *bona fide* schizophrenia risk gene.

**BOX. *ZNF804A* – the prototypical schizophrenia gene and its implications for psychopharmacology.**

*ZNF804A* (zinc finger protein 804A) illustrates the questions which arise, the approaches which are being taken, and the progress which is being made, when investigating the biological and therapeutic implications of a schizophrenia risk gene.

A SNP, rs1344706, within *ZNF804A* was the first to show good evidence for genome-wide association to schizophrenia (*p*=1.61×10^-7^), in a sample of 7300 cases and 12,800 controls (O’Donovan et al., 2008). The SNP is a common A/C (also denoted T/G) polymorphism, with A being the risk allele. The finding was confirmed in a meta-analysis of 19,000 cases and 38,000 controls with an overall *p*-value of 2.5×10^-11^, becoming 4.1×10^-13^ when bipolar disorder subjects were included (Williams et al., 2011). The odds ratio is about 1.10. But beyond these statistics, knowing the significance of the finding for understanding and treating schizophrenia requires several questions to be answered, including: what does *ZNF804A* do? What are the functional correlates of rs1344706 and how does the genotype affect disease risk?

*ZNF804A* is so-called because it is thought to be a member of the zinc finger protein family of transcription factors, based on sequence homology (a Cys-Cys-His-His [C2H2] motif) in one part of the gene (Razin et al., 2012). However, at the time of its first association to schizophrenia, there was no direct evidence about what the gene does. Subsequent studies in cell culture have shown that the gene regulates expression of, and interacts with, some other genes, consistent with this role (Girgenti et al., 2012; Hill et al., 2012). In human brain, *ZNF804A* mRNA is expressed throughout life, peaking prenatally, and with localisation of the encoded protein mainly to cortical pyramidal neurons. The immunoreactivity is not limited to the nucleus (as might be expected were it acting solely as a transcription factor), but also seen in the cytoplasm, suggesting additional roles (Tao et al., 2014). A combination of methods (RNA-seq and 5’-RACE) show that, as well as the canonical transcript (encoded by the four known exons of the gene), a truncated isoform is also abundant in human brain (Tao et al., 2014).

These initial findings about the biology of *ZNF804A* are complemented by efforts to reveal the biology (and thence disease risk) attributable to rs1344706-indexed genetic variation. Since the SNP lies in an intron, it cannot affect the amino acid composition of the protein itself. One possibility is that the genetic signal arises from a causal, coding (‘non-synonymous’) genetic variant for which rs1344706 is a proxy; however, no such variants have been found (Dwyer et al., 2010). Thus, rs1344706, and/or other non-coding SNPs with which it is in linkage dysequilibrium, are likely the source of the genetic signal. Indeed, the PGC2 meta-analysis finds additional SNPs within *ZNF804A* which are also genome-wide significant (Schizophrenia Working Group of the Psychiatric Genomics Consortium, 2014). The most plausible mechanism for the association is that one of the SNPs, or a haplotype containing them, affects regulation of the gene – that is, its expression (Cooper, 2010) – and this in turn modifies, in some way, the function of *ZNF804A*, and potentially that of its downstream and interacting biochemical targets. One strategy to investigate whether rs1344706 (or other SNP) alters *ZNF804A* expression is to compare *ZNF804A* mRNA abundance between those with and without the risk allele, or see if the transcript is differentially expressed from the two alleles in heterozygotes (using an expressed proxy SNP). Several such studies have been done in adult human brains, and no consistent picture has emerged (Guella et al., 2014; Hill and Bray, 2012; Williams et al., 2011). However, in fetal brain, the risk allele is associated with lower *ZNF804A* mRNA expression (Hill and Bray, 2012). In a refinement of this observation, Tao et al. (2014) confirmed that rs1344706 impacted on *ZNF804A* expression in fetal but not adult brain, but also showed that the effect was limited to the truncated transcript mentioned above, not influencing full-length *ZNF804A* mRNA. A molecular hypothesis for the pathogenic role of rs1344706 is therefore that it modulates the relative abundance of the two *ZNF804A* mRNAs during prenatal brain development. As well as needing replication, a finding of this kind begs further questions, including: Why does this difference matter? What are the two isoforms doing? Testing this will be a challenge. Hill and Bray (2011) show that rs1344706 affects protein nuclear binding, which may be one downstream effect of the allelic difference in expression, and Hess and Glatt (2013) discuss other potential molecular correlates of the risk allele.

In parallel with the molecular studies, several groups have investigated the functions of rs1344706 and *ZNF804A* at a systems level, using neuroimaging. These studies indicate that rs1344706 does not influence brain volumes (Cousijn et al., 2013) but may affect neural connectivity (e.g. Cousijn et al., in submission; Esslinger et al., 2009; Rasetti et al., 2011) and cortical functioning (e.g. del Re et al., 2014; O’Donoghue et al., 2014). Parenthetically, *ZNF804A* shows association to schizophrenia in Chinese populations, but to a different SNP, rs1366842 (Li and Su, 2013); this SNP is coding, and thus may have a different mechanistic basis for its disease association compared with rs1344706.

In summary, *ZNF804A* illustrates that a statistically compelling genetic association to schizophrenia risk does not, in itself, lead to any immediate functional understanding nor therapeutic implications. It also highlights that revealing the relevant biology of the gene and the mechanism of risk – and hence to determine the potential of the gene as a drug target – is complex, and requires multifaceted and extensive investigations.

### CNVs

Some of the genetic risk for schizophrenia is not mediated via common SNPs but by CNVs and other rare variants. CNVs are lengths of DNA (of the order of a million nucleotides) which are either deleted or duplicated, but which are too small to have been seen using conventional karyotyping methods. Recent microarray and other technologies show that CNVs are a normal feature of the genome, but also that CNVs which affect particular genomic regions are associated with an increased risk of schizophrenia. Eight such genomic regions are well established (Mowry and Gratten, 2013). Most encompass multiple genes, although two affect a single gene: 2p16.3 deletion (*NRXN1*; neurexin 1), and 7q36.3 duplication (*VIPR2*; vasoactive intestinal peptide receptor 2). Compared with schizophrenia-associated SNPs, each CNV is penetrant (Kirov et al., 2014) and confers a significantly increased risk of illness (odds ratios for several CNVs exceed 8), but each CNV is extremely rare, except for hemideletion of 22q11 (velocardiofacial syndrome; Schneider et al., 2014). The contribution that CNVs make overall to the aetiology of schizophrenia is unknown; a recent population-based study estimated that 5% of cases had a CNV of probable causal significance (Costain et al., 2013), though other estimates are lower (Rees et al., 2014). Many schizophrenia-associated CNVs occur *de novo* (Xu et al., 2011); the others are inherited. Like SNPs, the schizophrenia-associated CNVs do not show diagnostic specificity, also conferring risk of autism and learning disability (Malhotra and Sebat, 2012), though perhaps not bipolar disorder (Grozeva et al., 2010).

The dichotomy presented here between common SNPs of small effect, and rare but penetrant large CNVs, is an over-simplification. Exome sequencing (in which the expressed regions of each gene are sequenced in their entirety) is revealing a spectrum of schizophrenia-associated genetic variants (Gilman et al., 2012; Gulsuner et al., 2013), as there is with other psychiatric disorders (Visscher et al., 2012). In a recent large study, Purcell et al. (2014) show that schizophrenia is also associated with rare single nucleotide coding variants and by small insertions or deletions affecting a few nucleotides (‘indels’). This form of genetic variation was previously largely invisible. Purcell et al. (2014) estimate that such mutations account for a broadly comparable proportion of schizophrenia risk as do CNVs, with both contributing roughly one-tenth of the heritability attributable to common SNPs. Fromer et al. (2014) use family trios to show that these mutations are commonly *de novo*, and associated with more neurodevelopmental and cognitive impairment than cases without such mutations. It is important to note that these rare variant studies do not implicate conclusively any specific gene, but instead reveal an overall (and very modest) excess of such variants in schizophrenia, with clustering to functionally defined gene networks, consistent with the GWAS findings, as described below. Note also that, as with SNPs, proving the causality of a disease-associated rare variant is not a trivial undertaking (MacArthur et al., 2014).

There is debate as to whether research should focus on the genes and SNPs implicated by GWAS, since they are common, or on the rare variants, since their effects are penetrant and thus provide more traction on the core biology. Given the proliferation of genetic findings, only a selected few (in either category) can be taken forward for experimental investigation. Different research groups are making different choices and adopting different strategies, and it remains to be seen which prove to be most successful (McCarthy et al., 2014).

## The ‘missing heritability’

The existing findings account for only a minority of the heritability of schizophrenia, and there are various explanations for what accounts for the rest (Gibson, 2012; Lee et al., 2011; Manolio et al., 2009). Firstly, as noted, many more SNPs, and more CNVs and rare variants, will no doubt be found as exome- and genome-sequencing studies bear fruit. Secondly, it is likely that at least some, and possibly much, of the genetic risk reflects gene–gene interactions (epistasis; Mackay, 2014) rather than simply the cumulative effect of multiple independent genes (Phillips, 2008). There are preliminary clinical (Nicodemus et al., 2010), and experimental (Papaleo et al., 2014) data which support a role for epistasis in schizophrenia, but it has not been systematically investigated, and GWAS studies are not well suited to identify it. Thirdly, epigenetic factors (such as DNA methylation and histone modifications) may be involved (Daxinger and Whitelaw, 2012; Dempster et al., 2013), and contribute to gene–environment interactions, whereby part of the genetic risk for schizophrenia operates by altering sensitivity to environmental factors, such as obstetric complications or early use of cannabis. As with epistasis, there are some intriguing findings (e.g. Børglum et al., 2014; Di Forti et al., 2012; Nicodemus et al., 2008) but as yet few robust data (Iyegbe et al., 2014).

## From genes to networks and pathways

For all forms of schizophrenia-associated genetic variation, there is increasing evidence, both empirical and bioinformatic, that the implicated genes converge upon biochemical pathways and networks. This is an important finding, both for understanding the core pathophysiology of the disorder, and also for therapeutics. Five examples are mentioned here.

### NMDA receptor signalling

A notable convergence is upon NMDA receptor (NMDAR) signalling, providing a genetic complement to, and corroboration of, the prominent pre-existing hypothesis that NMDAR hypofunction (and glutamate synaptic function more generally) is important in the pathophysiology of schizophrenia (Coyle et al., 2003; Frohlich and Van Horn, 2014; Harrison and Eastwood, 1998; Kantrowitz and Javitt, 2010; Marek et al., 2010; Olney and Farber, 1995). The initial suggestions for a genetic convergence on NMDAR signalling were based on candidate gene findings (Collier and Li, 2003; Harrison and Owen, 2003; Harrison and Weinberger, 2005; Moghaddam, 2003) and received preliminary support from a bibliometric analysis (Harrison and West, 2006). Much stronger evidence has followed. NMDAR-related and postsynaptic signalling complex genes are over-represented amongst schizophrenia-associated CNVs (Kirov et al., 2012) and are also enriched for rare variants (Fromer et al., 2014; Purcell et al., 2014; Timms et al., 2013). Finally, a SNP within the NMDAR *GRIN2A* subunit gene is now genome-wide significant for schizophrenia, as are SNPs at the loci for *GRIA1, GRM3* and *SRR* (Table 1), all of which impact on NMDAR signalling. Moreover, NMDAR signalling is critically involved in synaptic plasticity, interacting with the activity-regulated cytoskeletal protein (ARC) complex. It is thus notable that schizophrenia genes are also enriched for ARC genes (Fromer et al., 2014; Glessner et al., 2010; Lips et al., 2012; Malhotra et al., 2011; Purcell et al., 2014) and for those involved in other aspects of synaptic transmission (Kenny et al., 2014; Lips et al., 2012; Owen et al., 2005; Schizophrenia Working Group of the Psychiatric Genomics Consortium, 2014). In total, there is now compelling evidence that synaptic dysfunction, particularly that related to NMDAR signalling, is one of the pathways by which the genetic predisposition to schizophrenia is mediated (Pocklington et al., 2014).

### Immune function and the MHC locus

An immune involvement is another longstanding hypothesis of schizophrenia, based on various lines of evidence (Carter et al., 2014; Patterson, 2009), including an association with human leukocyte antigen (HLA) status (McGuffin, 1979). GWAS studies have confirmed that the MHC locus on chromosome 6, which encodes HLA and other immune genes (as well as some genes not related to immune function), is associated with the disorder (Corvin and Morris, 2014; McGuffin and Power, 2013; Purcell et al., 2009; Schizophrenia Working Group of the Psychiatric Genomics Consortium, 2014). There is also evidence for involvement of immune genes located outside the MHC region (Schizophrenia Working Group of the Psychiatric Genomics Consortium, 2014). The MHC locus is probably one of the sources of genetic difference between schizophrenia and bipolar disorder, since the latter shows no association (Andreassen et al., 2013). Intriguingly, the discovery that MHC genes also have functions in brain development and in glutamate receptor signalling and synaptic plasticity (Fourgeaud et al., 2010; Lee et al., 2014) provides a potential link between MHC- and NMDAR-related aspects of the genetic aetiology of schizophrenia (McAllister, 2014).

### Calcium signalling

Calcium signalling is emerging as another genetic convergence. *CACNA1C*, which encodes the L-type calcium channel Ca_v_1.2 α subunit, was first shown to be genome-wide significant for bipolar disorder (see Bhat et al., 2012). It was subsequently shown to also be a GWAS hit across several disorders (Cross Disorder Group of the Psychiatric Genomics Consortium, 2013a) and significant for schizophrenia alone, along with *CACNB2* (encoding the Ca_v_β2 subunit) and other genes involved in calcium regulation (Ripke et al., 2013; Schizophrenia Working Group of the Psychiatric Genomics Consortium, 2014). These genes also contain an excess of rare variants in schizophrenia (Purcell et al., 2014). Abnormalities of calcium signalling were already well documented in bipolar disorder (Casamassima et al., 2010), but involvement in schizophrenia was perhaps less anticipated. Of note, calcium signalling (Berridge, 2014), including L-type calcium channels, are integral to many aspects of synaptic plasticity, key signalling cascades, and cognition (Heck et al., 2014; Moosmang et al., 2005; White et al., 2008).

### NRG1–ERBB4–PI3K–AKT1 pathway

The above three examples concern over-representation of genes within a functionally defined gene set. Complementing these, the best example of convergence of multiple genetic hits within a well-established biochemical pathway is the neuregulin 1 *(NRG1)–ERBB4–PI3K–AKT1* pathway. Although it is important to point out that none of these genes are significant in the large GWAS studies, there is evidence for association of all four genes to schizophrenia, and for epistasis between them (Emamian et al., 2004; Harrison and Law, 2006; Hatzimanolis et al., 2013; Law et al., 2012; Nicodemus et al., 2010; Norton et al., 2006). This is a key pathway regulating cellular growth and activity (Mei and Nave, 2014; Vanhaesebroeck et al., 2010; Zheng et al., 2012). Its role in schizophrenia illustrates the point made earlier that the risk SNPs may preferentially affect certain isoforms of each gene; for example, type IV NRG1 (Law et al., 2006; Paterson et al., 2014; Tan et al., 2007), the CYT-1 isoform of *ERBB4* (Law et al., 2007), and the p100δ isoform of the catalytic subunit of *PI3K* (Law et al., 2012). This isoform selectivity is not only pathophysiologically interesting, but may provide opportunities for selective drug targeting (Barrie et al., 2012; Lipscombe et al., 2013). Furthermore, *NRG1* and *ERBB4* have direct and indirect interactions with NMDAR signalling (Banerjee et al., 2010), whilst *AKT1* (protein kinase C) impacts on the GSK3β–Wnt pathway which is also implicated in the disorder (Freyberg et al., 2010). These wider interactions highlight that there may be ‘meta-convergence’ of schizophrenia genetic risk across quasi-independent pathways.

### MIR137 and its targets

A different form of genetic convergence is related to the microRNA-encoding gene *MIR137*, another locus which is genome-wide significant for schizophrenia. MicroRNAs are non-protein-coding genes, whose RNA products bind to the 3’ region of specific mRNAs and inhibit their translation. Several of the mRNA targets of *MIR137* (determined empirically, or predicted bioinformatically) are also GWAS schizophrenia genes (including *TCF4, ZNF804A*, and *CACNA1C*; Kim et al., 2012; Kwon et al., 2013; Wright et al., 2013; see also Boudreau et al., 2014), suggesting that there may be functional impairment of a network of *MIR137*-regulated genes in schizophrenia. However, the evidence is limited, and it is not even certain that the signal at this locus is attributable to *MIR137* and not to the adjacent *DYPD* gene – an illustration of the difficulty noted earlier moving from locus to gene (Schizophrenia Working Group of the Psychiatric Genomics Consortium, 2014).

## Therapeutic implications of genetic discoveries

Genomic discoveries can contribute to therapeutics in several ways (Green and Guyer, 2011; Manolio, 2013): target identification; rational drug design; genetic stratification in clinical trials; genetic prediction of efficacy and toxicity; development of gene therapy; and even genetic influences on the outcome of psychological and social interventions. Here, the discussion focuses on how recent and forthcoming genetic discoveries about schizophrenia can help to identify drug targets to treat the disorder. Genetic effects on response to, and side-effects of, existing treatments are discussed elsewhere (Arranz et al, 2011; Harrison, 2014; Zhang and Malhotra, 2013).

Whilst genetics provides the rational route to treatments which can correct the core underlying biochemical abnormalities of a heritable disorder like schizophrenia, such benefits are unlikely to be either direct or rapid. A reality check is provided by Plenge et al. (2013), who list a series of generic criteria to be considered when applying genetic findings to drug target validation (Table 2). Whilst it is a moot point whether these criteria are all valid in the context of schizophrenia, they do serve to illustrate the substantial size of the task ahead. Indeed, at present we fall at the first hurdle (and at most if not all of the others); for example, we have few if any causal variants, we merely have statistically associated tag SNPs and large CNVs, the impacts of which (as well as the relevant functions of the affected gene[s]) are at best poorly understood. Thus, it is usually not clear whether the therapeutic goal would be to enhance or decrease the actions of the implicated gene product. And, critically, since each gene independently contributes such a small amount of the variance, a drug may well not produce significant therapeutic traction even if it were perfect at correcting the abnormality.

**Table 2.** Criteria relevant to prioritising drug targets based on genetic findings.

1. The gene contains a causal variant unequivocally associated with the disorder.
2. The biological function of the gene, and the causal variant within it, are known.
3. The gene harbours multiple causal variants of known biological function.
4. The gene has a gain-of-function allele that protects against the disorder, or a loss-of-function allele that increases risk.
5. The gene must be related to the clinical indications targeted for treatment.
6. The genetic variant is associated with an intermediate phenotype that can serve as a biomarker.
7. The gene is ‘druggable’.
8. The genetic variant is not associated with other, adverse, phenotypes.
9. Corroborating biological data support the genetic findings.

The criteria are adapted from Plenge et al. (2013), listed in their order of priority.

So, what is the way ahead, avoiding both naïve optimism and nihilism? The middle-ground is to use genetic information not as a stand-alone determinant for target identification or validation, but as one factor to be considered in conjunction with a range of other sources of information, and taking into account practical considerations. How might this work, and what are some of the issues involved (see Table 3)?

1. Genes which were already drug targets for schizophrenia, or which have a direct effect on a current drug target, are in a unique category, since in this instance the fact that they may contribute to the genetic aetiology of the disorder merely augments the prior considerations which had led them to be of interest. There are now GWAS-significant genes in this category, including *GRM3* and *SRR. GRM3* encodes mGlu3; a mGlu2/3 agonist was already in clinical trials, albeit ultimately with disappointing results. *SRR* encodes serine racemase, the enzyme which synthesises D-serine, which has shown positive results in clinical trials augmenting antipsychotics (Moghaddam and Javitt, 2012).
2. *GRM3* and *SRR* also illustrate that some genes are inherently more attractive therapeutic targets than others. Genes encoding receptors, enzymes, ion channels, or transporters are more likely to be tractable than genes encoding transcription factors, non-protein-coding genes, or genes whose function is currently unknown. Similarly, genes predominantly expressed in fetal brain are less appealing as drug targets, other things being equal, than those abundantly expressed throughout adulthood (see Gulsuner et al., 2013; Xu et al., 2012).
3. Therapeutic interest in some genes and pathways is facilitated by the fact that they were already being targeted for other disorders. For example, the NRG1–ERBB4–PI3K–AKT1 pathway mentioned earlier is a cancer therapeutic target, with Herceptin (an anti-ERBB2 monoclonal antibody) used in breast cancer, and other ERBB and PI3K inhibitors under investigation (Fruman and Rommel, 2014). Indeed, a drug was already available which selectively inhibited the PI3K p110δ isoform implicated by Law et al. (2012), having been developed for leukaemia. When tested in relevant preclinical models, it showed effects predictive of antipsychotic efficacy (Law et al., 2012), as do some ERBB inhibitors (Mizuno et al., 2013). Clearly, there are many issues to consider before such compounds could be proposed for use in schizophrenia, but at least their availability for repurposing, and the prior knowledge about their effects and toxicity, has the potential to shorten development time.
4. As noted earlier, targeting individual genes may be of limited benefit. This is where the evidence for involvement of functionally related genes, networks or biochemical pathways (whether via epistasis, protein–protein interactions, or other mechanisms) becomes critical from a therapeutic perspective. If multiple risk genes do converge on a smaller number of protein hubs or biochemical pathways, then it becomes possible, in theory at least, to normalise the pathway function using a downstream target, regardless of which gene(s) underlie the abnormality in each patient. The leading example of this kind at present is the convincing genetic convergence on glutamate synapses and NMDAR-mediated signalling, which have long been a therapeutic target (reviewed in Field et al., 2011; Moghaddam and Javitt, 2012) because of the prior evidence and hypotheses about their role in schizophrenia. The increasing genetic evidence implicating calcium signalling in schizophrenia also has clear therapeutic implications; moreover, the established use of L-type calcium channel blockers in other medical conditions (Zuccotti et al., 2011) is also a relevant consideration. However, even though targeting downstream, convergent effects of risk genes is attractive, the question remains as to what kind of effect on the pathway is desired: enhancement, inhibition, stabilisation, etc. To date, such information is very limited.
5. Genetics may also facilitate more targeted treatment towards particular features or subtypes of schizophrenia, such as cognitive and negative symptoms. This includes genes which do not affect the risk of illness itself. For example, SNPs within the sodium channel gene *SCN2A* influence general cognitive ability, cortical efficiency, and gene expression in patients (and their unaffected siblings), but have no effect, or the reverse effect, in controls (Dickinson et al., 2014). This study highlights that genetics has more to offer schizophrenia therapeutics than simply identification of SNPs or genes which happen to show main effects in case-control comparisons, however large the samples. Equally, the realisation that many genes for schizophrenia confer risk for other disorders (Cross Disorder Group of the Psychiatric Genomics Consortium, 2013a,b) suggests that novel drugs impacting on these targets may be worth investigating across a similarly wide range of phenotypes.

**Table 3.** Questions affecting the therapeutic targeting of a schizophrenia risk gene.

Question	Comments
What is the genetic evidence?	How strong is the evidence? What kind of involvement – SNP, CNV, rare mutation? How big is the effect size? How common is the risk variant? With what other phenotypes is the gene associated?
What does the gene code for?	Is it protein-coding? If so, what class of protein (e.g. receptor, enzyme, transporter)?
What is known about the gene’s biology?	What are its main functions? Evidence from genetically modified mice or other experimental data? Where and when is the gene expressed and functional? Are there isoforms? Are there case-control differences in expression or function of the gene (independent of genotype)?
Are there functional differences associated with risk genotype?	Has the risk variant itself been identified? Is there a known effect on gene expression or function? If so, is it enhanced or decreased, and where and when does this occur?
Are interacting genes also implicated in schizophrenia?	Are there gene–gene, protein–protein, or other functional interactions with other schizophrenia risk genes? What is known about the resulting network/pathway in terms of its function or therapeutic potential?
What tools are available to investigate the function of the gene, or the difference between risk and non-risk forms?	Knockout, transgenic, or conditional genetically modified mice? Validated ligands or antibodies? Licensed compounds?

As might be expected, genetically driven therapeutic progress in medicine to date has been greatest in situations where a single gene is the sole or primary cause of the disease (e.g. for certain cancers and Mendelian disorders; Green and Guyer 2011; Sanseau et al., 2012). Yet even in these domains, advances have been few (Dietz, 2010). Within psychiatry, we still await mature therapeutic fruits of research into the neurodegenerative (e.g. Huntington’s disease; familial Alzheimer’s disease) and neurodevelopmental (e.g. Rett syndrome) disorders for which the causal genes and the mutations within them have been known for well over a decade, and despite considerable advances in understanding their molecular pathogenesis (Gadalla et al., 2011; Huang and Mucke, 2012; Ross and Tabrizi, 2011). On the other hand, these cautionary notes in no way detract from the significance of the genetic discoveries for schizophrenia therapeutics. It is from genetics that the core biochemical and molecular basis of the disorder will finally be elucidated, and it is from the latter understanding that rationally designed and effective treatments will be developed. This is a prize well worth striving for. Notably, a recent study showed how the genetics of rheumatoid arthritis is informing target validation and drug discovery. Okada et al. (2014) report that the targets of existing rheumatoid arthritis drugs significantly overlap with the disease-associated genes (3.7-fold enrichment, *p*<10^-5^); they also provide genetic data which suggest that certain drugs currently being used in cancer are worth trialling in rheumatoid arthritis. These findings are encouraging, since the genetic architecture of rheumatoid arthritis shows many similarities with schizophrenia (e.g. both currently have ~100 genome-wide significant loci, most with odds ratios <1.2, and without the causal variant at each locus having been identified).

## Conclusions

Genetic research over the past decade has provided fundamental insights into the nature of schizophrenia, with identification of the first indisputable risk loci and genes, and discovery that rare variants also contribute to the genetic predisposition. The results are beginning to reveal the key gene networks and biochemical pathways, are already driving the design and focus of neurobiological studies of the disorder, are influencing pharmacotherapeutic research strategies, and – though not discussed here – are close to impacting on clinical diagnostic practice (Costain et al., 2013; Rees et al., 2014). Equally, with the discoveries comes the sobering realisation that the genetic basis of schizophrenia is even more complex, in many ways, than had generally been anticipated. Finding loci and genes for schizophrenia is a triumph, but it is merely the start of a long process towards meaningful biological understanding, let alone better treatment, of the disorder. Genetics augments but does not replace the other key elements in drug development, and does not remove the many other hurdles (Filippich et al., 2013; Hyman, 2014; Pratt et al., 2012; Winchester et al., 2014). But at least the genetic findings provide a strong rationale for, and firm foundations on which to build, the next generation of studies, as we sequence rather than sample the genome, integrate genomics with the other ‘omics’, develop new analytical and bioinformatic tools, discover how genes interact with each other and with the environment, and clarify how the genes and their variants actually drive the pathophysiology. It is to be hoped that these opportunities encourage further academic and pharmaceutical investments. The potential benefits and rewards are huge – and put the scale, expense, and risk of the undertaking into proportion.
